# Do premolar extractions necessarily result in a flat face? No, when properly indicated

**DOI:** 10.1590/2177-6709.23.5.082-092.bbo

**Published:** 2018

**Authors:** Susiane Allgayer, Maurício Barbieri Mezomo

**Affiliations:** 1 Professor of Orthodontics at Associação Brasileira de Odontologia - Seção Rio Grande do Sul (Porto Alegre/RS, Brazil).; 2 Professor of Orthodontics at Centro Universitário Franciscano, Faculdade de Odontologia, (Santa Maria/RS, Brazil).

**Keywords:** Tooth extraction

## Abstract

The esthetic benefits are among the main goals of orthodontic treatment; therefore, tooth extractions have been avoided as a protocol for orthodontic treatment because they may impair the facial profile. The present article discusses aspects as the magnitude and response of soft tissue profile due to changes in incisor positioning, and the effect of different sequences of premolar extraction. One case report illustrates the subject, with favorable and stable esthetic and occlusal outcomes five years after orthodontic treatment with extraction of second premolars.

## Resumo

Os benefícios estéticos estão entre os principais objetivos do tratamento ortodôntico. Por isso, as extrações dentárias têm sido evitadas como protocolo de tratamento ortodôntico, pois podem prejudicar o perfil facial. No presente artigo, serão discutidos aspectos como a magnitude da resposta do perfil de tecidos moles em decorrência das mudanças na posição dos incisivos e os efeitos de diferentes sequências de extrações de pré-molares. Um caso clínico ilustrará o tema, com resultados estéticos e oclusais favoráveis e estáveis cinco anos após o tratamento ortodôntico com extrações de segundos pré-molares.

## INTRODUCTION

In recent years, there has been a noticeable increase in awareness and interest in facial esthetics.^1^ ^,^ ^2^ The esthetic benefits are among the main goals of orthodontic treatment,^3^ and clinicians are often asked about possible changes in the profile caused by the treatment.^4^ The fact that dental extractions may cause a flat face^4^ ^-^ ^7^ due to excessive incisor retraction has discouraged the orthodontists to adopt this treatment protocol. However, extractions can benefit the profile when properly indicated.^8^ ^-^ ^13^

Treating patients without extraction simply not to remove teeth or to simplify the treatment is not justified, because it may impair the result and stability of orthodontic treatment. The ideal approach is to apply the correct extraction protocol for each type of malocclusion.^6^ In other words, although non-extraction treatment has become popular, many orthodontic patients have some shortage of space or crowding requiring extractions for a favorable treatment outcome.^10^ ^,^ ^14^

There is general agreement that orthodontic treatment can influence the facial profile, but there is still disagreement on the magnitude of soft tissue response as a consequence of changes in tooth position and alveolar process. Moreover, there are contradictory opinions about the facial profile changes when different sequences of premolar extractions are analyzed.^8^ ^,^ ^11^ ^,^ ^15^ ^-^ ^21^Some investigators^22^ ^-^ ^24^suggest more studies to define the effects of the different premolar extraction sequences, as well as to quantify the cumulative effect of aging after this treatment approach on the facial profile.^10^

Therefore, this paper discusses aspects as the magnitude and response of the soft tissue profile as a consequence of changes in incisor positioning, and the effect of different sequences of premolar extraction. One case report illustrates the subject, with favorable and stable esthetic and occlusal outcomes 5 years after orthodontic treatment with extraction of second premolars.

## CASE REPORT 

**Figure 1:**
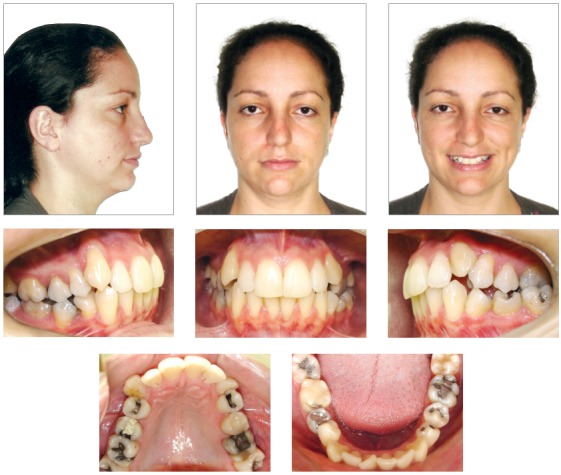
Pretreatment facial and intraoral photographs.

**Figure 2:**
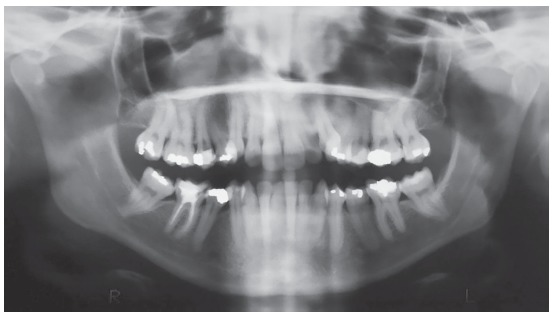
Initial panoramic radiograph.

A female patient, aged 27 years and 4 months, had the chief complaint about the “esthetic appearance of her teeth”. She reported to be ashamed of smiling because of the high and rotated maxillary canines. The facial photographs showed proportional facial thirds and straight facial profile with a concave lower facial third. A deficient and asymmetric smile was evident due to the malposition of the maxillary canines. The intraoral photographs evidenced complete Class II molar and canine relationships, 4-mm overjet, 3-mm overbite, and negative tooth-size discrepancy of 5 mm in the maxillary arch and 8.5 mm in the mandibular arch, besides 2-mm deviation of the maxillary midline to the right side. The patient also exhibited crossbite on the left side (Fig 1). The panoramic radiograph exhibited all teeth except for the third molars. Also, there was significant horizontal bone loss for the age at the premolar region; endodontic treatment of tooth 46 and impairment of dental health due to large restorations in several teeth (Fig 2).

**Figure 3:**
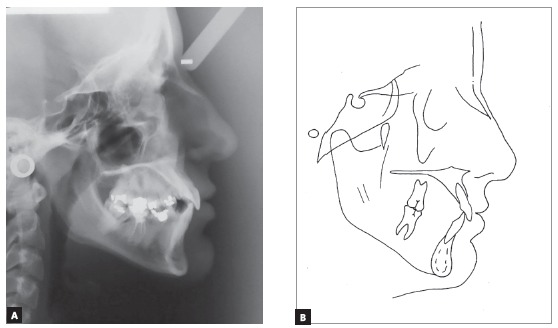
Initial lateral cephalogram (A) and tracing (B).

Cephalometric analysis revealed skeletal Class I relationship (ANB = 1^o^). Considering occlusal plane angle values (SN.Ocl = 33^o^), mandibular plane (SN.GoGn = 42^o^) and Y axis (Y-axis = 62^o^), a hyperdivergent skeletal pattern prevailed. The maxillary incisors were buccally tipped and protruded (1-NA = 9 mm and 1.NA = 26^o^) and the mandibular incisors were well positioned (1-NB = 6 mm and 1.NB = 25^o^, IMPA = 90^o^). The upper lip was retruded in 4 mm and the lower, in 2 mm in relation to the S line (Fig 3 and Table 1). 

**Table 1:** Initial (A) and final (B) cephalometric values

	Measurements		Normal	A	B	A/B diff.
Skeletal pattern	SNA	(Steiner)	82°	73°	74°	1
	SNB	(Steiner)	80°	72°	72°	0
	ANB	(Steiner)	2°	1°	2°	1
	Wits	(Jacobson)	♀ 0 ±2 mm ♂ 1 ±2 mm	- 8	0	8
	Angle of convexity	(Downs)	0°	1°	2°	1
	Y-axis	(Downs)	59°	62°	62°	0
	Facial angle	(Downs)	87°	83°	84°	1
	SN-GoGn	(Steiner)	32°	42°	41°	1
	FMA	(Tweed)	25°	33°	33°	0
Dental pattern	IMPA	(Tweed)	90°	90°	92°	2
	1.NA (degrees)	(Steiner)	22°	26°	20°	6
	1-NA (mm)	(Steiner)	4 mm	9mm	5mm	4
	1.NB (degrees)	(Steiner)	25°	25°	29°	4
	1-NB (mm)	(Steiner)	4 mm	6mm	6mm	0
	- Interincisal angle	(Downs)	130°	128°	129°	1
	1-APo	(Ricketts)	1 mm	5mm	5mm	0
Profile	Upper lip - S-line	(Steiner)	0 mm	- 4mm	- 4mm	0
	Lower lip - S-line	(Steiner)	0 mm	- 2mm	- 2mm	0

## TREATMENT PLAN AND APPLIED MECHANICS

The treatment objectives were to obtain normal occlusion, adequate overjet and overbite, correct the crowding and axial inclinations of maxillary anterior teeth, thus improving function, facial esthetics and smile characteristics. The treatment options were: extractions of four first premolars, extractions of maxillary first premolars and mandibular second premolars, extractions of the four second premolars, or the utilization of temporary anchorage devices to distalize the mandibular posterior teeth.

It was decided to extract the second premolars to avoid incisor retraction and undesirable change in the facial profile.

**Figure 4:**
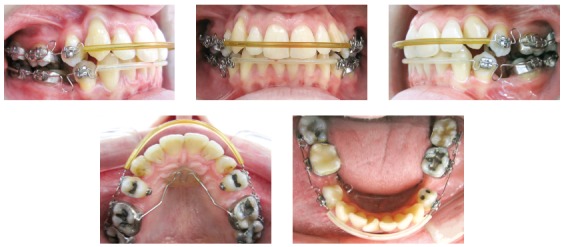
Intermediate intraoral photographs with Tweed teardrop loops archwire.

**Figure 5:**
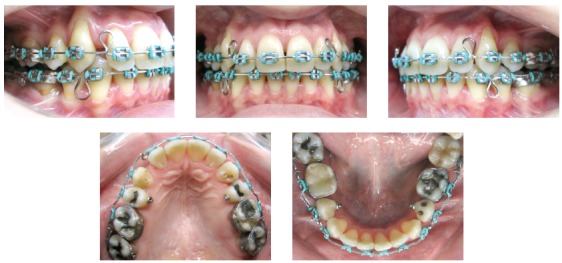
Intermediate intraoral photographs with retraction archwire.

Initially, Edgewise brackets with 0.022 x 0.028-in slot were placed on the molars and first premolars (3M Unitek, Monrovia, CA) and a Nance button for anchorage. An archwire with “teardrop loops”^25^ ^,^ ^26^ was placed in the edentulous space and also on the mesial surface of the first premolars in both arches, to retract these teeth to the extraction spaces (Fig 4). Following, brackets were bonded on the canines, which were retracted with Tweed multiloop archwire.^25^ ^,^ ^26^ After that, brackets were bonded on the incisors for alignment and leveling with 0.0175-in coaxial archwire and 0.014-in to 0.020-in stainless steel archwires. The mechanics for incisor retraction was applied with a 0.019 x 0.025-in “vertical closing loops archwire” with 6-mm vertical loops for final incisor retraction, which was completed in 8 months (Fig 5). Mounting was performed in a Bio-Art semi-adjustable articulator (Bio-Art Equipamentos Odontológicos Ltda, São Carlos/SP, Brasil), to check the interdigitation, intercuspation on the palatal aspect and protrusion and lateral guidances. The rebonding of some brackets was guided by the panoramic radiograph, as well as maxillary re-leveling, followed by 0.019 x 0.025-in stainless steel archwires with ideal torques. Posterior vertical intermaxillary elastics were applied for intercuspation and finalization. The total treatment time was 36 months.

## OBTAINED RESULTS

**Figure 6:**
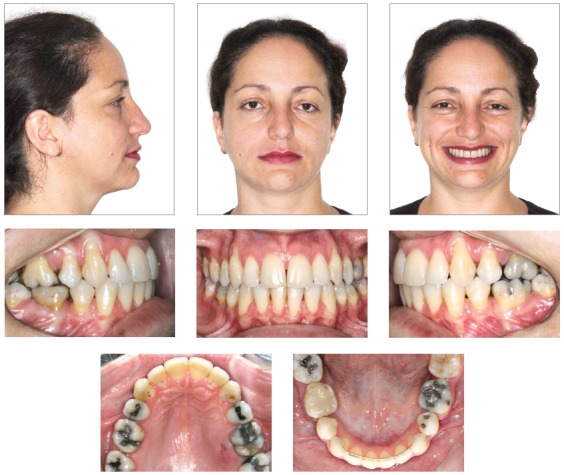
Final facial and intraoral photographs.

The posttreatment photographs confirmed that the extraction of premolars did not impair the profile. The adequate alignment and torque of anterior teeth provided support to the upper lip, enhancing the esthetics of the profile. The adequacy of tooth-size discrepancy and alignment filled the buccal corridor, providing greater amplitude, youth and attractiveness to the smile (Fig. 6). 

Analysis of the intraoral aspect evidenced Class I occlusion with ideal overjet and overbite, correction of crowding, coincident dental and facial midlines and solid interdigitation between the dental arches, including the second molars. 

**Figure 7:**
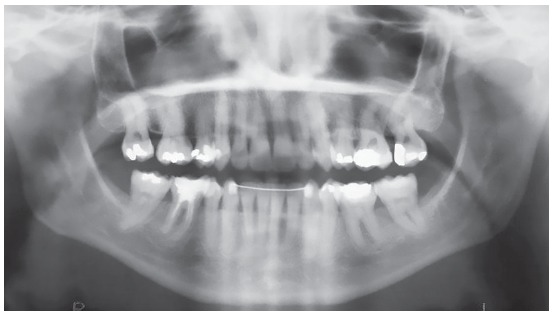
Final panoramic radiograph.

Adequate arch shapes were achieved, with teeth well positioned in the dental arches and maintenance of intercanine distance in the mandibular arch. The treatment also provided functional occlusion and good periodontal health, despite the gingival recessions. The determining factors of equipotent simultaneous bilateral contacts and immediate disocclusion in mandibular movements were established (Fig 6). The panoramic radiograph evidenced good root parallelism and integrity of dental roots (Fig 7).

**Figure 8:**
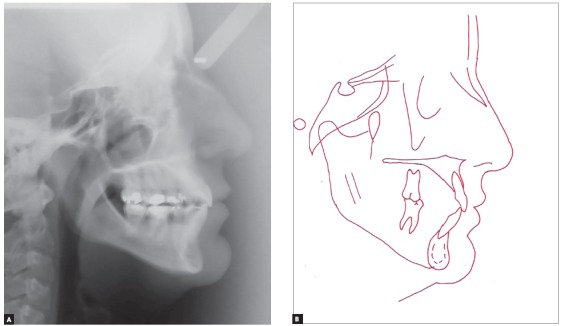
Final lateral cephalogram (A) and tracing (B).

The posttreatment cephalogram and superimpositions illustrate the changes achieved by treatment, evidencing slight changes in the nose and pogonion. The relationship between the lips and the Steiner’s S line remained unchanged, without damage to the profile (Upper lip to S Line = - 4 mm, Lower lip to S Line = - 2 mm). Also, the convexity angle increased 1^o^, despite the extractions. The applied mechanics did not yield undesirable mandibular rotation or mandibular plane opening (Fig 8 and Table 1).

**Figure 9:**
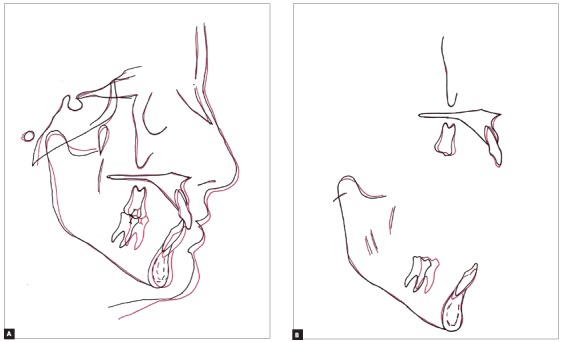
Total (A) and partial (B) superimpositions of initial (black) and final (red) cephalometric tracings.

The maxillary first molars were upright, remaining in the original position. There was uprighting of maxillary incisors and correction of torques, maintaining satisfactory inclination. The mild extrusion enhanced the exposure of maxillary teeth at rest and during smiling. The remodeling of point A, which was advanced in 1 mm, enhanced the support to the upper lip without flattening the profile (Fig 9A). There was marked mesial movement of mandibular first molars combined with more anterior mandibular positioning, which contributed to correct the molar relationship to Class I. The intrusion of mandibular incisor corrected the moderate overbite, which is compatible with extrusion of the maxillary incisor. The IMPA had slight variation from 90^o^ to 92^o^, indicating that the incisor remained well-positioned in relation to the mandibular plane (Fig 9B and Table 1). 

**Figure 10:**
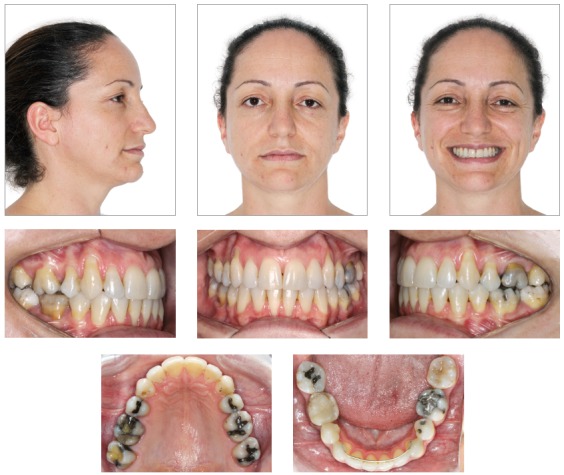
Final facial and intraoral photographs at 5-year follow-up.

The follow-up 5 years after completion of the active treatment stage revealed stable outcomes, accommodation of teeth allowing better occlusal relationship, and closure of small diastemata that were still present upon appliance removal. Also, the gingival recessions have remained stable (Fig 10).

## DISCUSSION

The success of orthodontic treatment depends on the careful analysis of all diagnostic elements and establishment of a correct treatment planning. Among the several decisions, the professional should determine if the success of intervention requires dental extractions.^2^ ^,^ ^10^ ^,^ ^13^ ^,^ ^15^ The extractions with orthodontic purpose, for correction of tooth crowding or intermaxillary discrepancies, have been controversial since the concepts of normal occlusion were initially enhanced, in the early 20^th^ century.^3^ ^,^ ^11^ ^,^ ^16^ ^,^ ^17^ ^,^ ^27^ ^-^ ^29^ Any tooth may be extracted, depending on each case, to provide more satisfactory esthetic and functional outcomes. Within this sense, there is consensus that the planning of orthodontic treatment should be customized.^14^ ^,^ ^24^ ^,^ ^30^

Based on these principles, the diagnosis of the need of adequacy of tooth size to the dental arches in this adult patient revealed the need of extractions. Correction of this malocclusion with extractions could have been a problem, especially for a patient with concave profile and thin upper lip. The weakened crown and need of endodontic treatment in the maxillary right second premolar, combined to the fact that the extraction of first premolars may excessively retract the facial profile, led to indication for extraction of second premolars.^26^

This extraction pattern resulted in a stable relationship between upper and lower lips and the Steiner’s S Line (Upper lip to S Line = - 4 mm, Lower lip to S Line = - 2 mm). The same result was presented by James^6^, who concluded that the extractions of maxillary and mandibular second premolars do not change the lower lip positioning in relation to the facial esthetic line of Ricketts.^31^ ^,^ ^32^

Tooth extractions might increase the profile concavity; however, the selected sequence of extractions, remodeling of point A of 1 mm and the mild extrusion of maxillary incisors provided greater support to the lips, enhancing the exposure of maxillary teeth at rest and during smiling. The extractions of second premolars allowed better control of incisors and of the lip retraction, avoiding the marked concavity of the facial profile that occurs after extractions of first premolars.^11^ ^,^ ^12^ Indicated in cases with moderate shortage of space, in individuals with balanced facial contours and well-positioned incisors in their dental arches, the extraction of second premolars is justified in the literature.^11^ ^,^ ^12^

Nance^11^ indicated the extraction of maxillary first premolars and mandibular second premolars in borderline cases with mild biprotrusion, in which the extractions of first premolars may excessively retract the facial profile. This was later corroborated by other investigators.^16^ ^,^ ^17^ ^,^ ^33^ James^6^ and Dewel^3^ ^,^ ^28^ described the moderate space deficiency, which is characteristic of borderline cases in individuals with balanced facial contours, as one of the basic diagnostic requirements for indication of extractions of second premolars. According to Carey,^29^ better results were achieved when malocclusions with discrepancies between 2.5 and 5 mm were treated by extraction of second premolars. However, according to Schoppe,^34^ the main indication included cases with discrepancies up to 7.5 mm, in individuals with muscular balance, proportional facial contour and incisors well-positioned in the dental arches. Confirming these findings, Castro^12^ described the advantages of extraction of second premolars for cases with need of extractions, especially for patients with satisfactory profile and favorable mandibular growth. 

Conversely, some authors did not observe direct correlation between the tooth to be extracted and lip positioning. However, they agree that the pretreatment and growth characteristics lead to different facial outcomes.^18^ ^-^ ^21^

In the present case, the effects of the adopted mechanics benefited the soft tissue profile of this patient. The uprighting of anterior teeth without retraction was especially important to maintain the upper lip support, which could have been a problem if the therapeutic approach had neglected the initial concave profile of the patient. According to Burstone et al,^35^ ^,^ ^36^ many factors affect lips position, including several orthodontic and surgical procedures. A good position of the lip can be obtained by surgically or orthodontically protruding incisors, increasing/reducing the chin prominence, or both.^8^ Specifically, in the present case, the Tweed teardrop archwire was selected, associated to the Nance button for anchorage control, for retraction of first premolars and canines. The anterior teeth were bonded and included at a later moment to protect the profile, which is demonstrated in the superimpositions. Also, according to Legan et al,^36^ the controlled retraction of mandibular incisors associated to the extrusion of maxillary teeth maintain the lip support, as in the present case.

Different from the findings of Herzog et al,^2^ the mandibular intercanine distance was maintained, assuring stability of the achieved results 5 years after treatment.^37^ ^,^ ^38^

## CONCLUSION

Although many innovative techniques have emerged over the past few years, this paper evidences that traditional treatment is still an excellent alternative, providing lasting results for shortage of space or crowding. The extractions of second premolars may assure the profile integrity when the challenge is to achieve space in cases of negative tooth-size discrepancy.

The professionals should be aware of the diagnosis and planning of the ideal pattern of tooth extractions, to achieve the esthetics of the profile and facial balance, as well as functional occlusion and stability. 
